# Genetic and non-genetic long-term trends of 12 different crops in German official variety performance trials and on-farm yield trends

**DOI:** 10.1007/s00122-014-2402-z

**Published:** 2014-10-12

**Authors:** Friedrich Laidig, Hans-Peter Piepho, Thomas Drobek, Uwe Meyer

**Affiliations:** 1 Bundessortenamt, Osterfelddamm 80, 30627 Hannover, Germany; 2 Bioinformatics Unit, Institute of Crop Science, University of Hohenheim, Fruwirthstrasse 23, 70599 Stuttgart, Germany

## Abstract

*****Key message***:**

**Yield progress in major German crops is generated mostly due to genetic improvement over the last 30 years. Comparison of trial-station with on-farm yield reveals considerable gaps that are widening over time.**

**Abstract:**

Yield progress of newly released varieties for 12 crops from official German trials over the period 1983 until 2012 was analysed to assess their value for cultivation and use (VCU). We paid special attention to dissect progress into a genetic and a non-genetic (agronomic) trend in order to quantify the contribution made by new varieties and by agronomic factors. In this study, we apply mixed models including regression components for genetic and agronomic trends. Ageing effects, depending on the difference of the actual testing year and the first year of testing of a particular variety, were estimated from the difference of fungicide and non-fungicide-treated trial pairs. Significant yield losses were found in all cereal crops due to assumed ageing effects. We compared national on-farm with official VCU trial yields with particular focus on whether gaps are widening over time. Results indicated a significant widening over time. In order to facilitate comparisons of results across crops, we calculated percent rates based on 1983 yield levels obtained from regression estimates. Most of the yield progress was generated by genetic improvement, and was linear showing no levelling-off. Ageing and selection effects need to be taken into account, because they may lead to overestimation of genetic trends. This study showed that contribution of agronomic factors is of minor importance in overall yield progress.

**Electronic supplementary material:**

The online version of this article (doi:10.1007/s00122-014-2402-z) contains supplementary material, which is available to authorized users.

## Introduction

Newly bred varieties must be evaluated for their value of cultivation and use (VCU) before they can be registered in the national list and released for commercial production. Important performance traits are yield, quality and disease resistance. Each year about 900 varieties from more than 30 different agricultural species enter trials to be tested for 2–3 years at up to 25 locations spread over the individual crop’s typical growing region in Germany. The number of varieties entering the first trial year ranges from just a few to more than 100, depending on the crop. However, only about 20 % of the candidate varieties are finally released.

Release of new improved varieties and their translation into farm production needs to be considered in the broader context of a worldwide growing demand for both food and energy. The current world population of about 7.2 billion—at 2013—is projected to reach about 9.6 billion in 2050, (United Nations World Population Prospects 2012). In developing countries an increased income per capita will raise demand and prices for crops due to higher consumption of calories and protein (Fischer et al. 2014). In the past 20 years there was a very small increase in world arable land where new land was nearly balanced by land loss (Fischer et al. 2014). The likely increase of arable land until 2050 will be about 10 % on the 2008–2010 annual average harvested area (Fischer et al. 2014). The minimum supply increase for staple crops (mainly wheat, rice, maize, and soybean) to 2050 would be 60 % (relative to 2010) in order to halve hunger (Fischer et al. 2014). This means that the minimum target for global yield increase for staple crops should be 1.1 % p.a. relative to 2012 yield (Fischer et al. 2014). Lobell et al. (2009) reported on the importance, magnitudes and causes of crop yield gaps around the world. They found a wide range of yield gaps, with average on-farm yields ranging from roughly 20–80 % of potential yield. According to Lobell et al. (2009) on-farm yield of about 80 % of potential yield may approximate the economic optimum level of production of major cropping systems. Reducing average on-farm yield gaps below 20 % of potential yield appears to be possible but only with modern crop management practices minimizing climatic risks and responding dynamically to changing soil, water and nutrient conditions (Lobell et al. 2009). With regard to those challenges the productivity of the available crop area has to be raised drastically in order to meet the need of a fast growing world population. Ewert et al. (2005) estimated a possible increase in crop productivity (crop production per land unit) of European agricultural land use of 25–163 % between 2000 and 2080 for winter wheat as reference variety by considering a wide range of changes in climate scenarios. They predicted that increases of productivity would exceed demand changes in Europe and identified technology development as the most important driver. Both improved varieties and crop management techniques need to be introduced into farm practice in order to cope with those deficiencies (Graybosch and Petersen 2010).

There are quite a few studies on yield trends published (e.g. Schuster 1997, 1977; Silvey 1978, 1981; Brancourt-Hulmel et al. 2003; Perry and D’Antuono 1989 and more recently, e.g. Peltonen-Sainio et al. 2009; Mackay et al. 2011; Kristensen et al. 2011; Brisson et al. 2010; Lin and Huybers 2012; Lopes et al. 2012; Rijk et al. 2013; Loel et al. 2014). However, comparisons of genetic and non-genetic trends need to be considered with caution. Cultivar interaction with agronomic practices and with biotic and abiotic environments should be taken into account as pointed out in Evans and Fischer (1999). Genetic progress under low input conditions tends to overestimate genetic trends (Brisson et al. 2010; Mackay et al. 2011; Piepho et al. 2014). Factors influencing the extent of genetic progress may change over time. Hence, it is useful to consider different periods separately if estimates are taken over a longer time interval (e.g. Schuster 1997; Peltonen-Sainio et al. 2009; Lillemo et al. 2010; Mackay et al. 2011). Genetic progress in Dutch crop yields (Rijk et al. 2013) for winter wheat, spring barley, potato and sugar beet over 30 years showed a linear genetic trend. Additionally, significant year effects were found for most crops in the Dutch study when corrected for genetic progress. In a similar UK study (Mackay et al. 2011), it is reported that for cereals and oil seed rape at least 88 % of the improvement in yield is attributable to genetic sources, whereas for forage maize and sugar beets, plant breeding and agronomy had contributed about equally. The impact of plant breeding on spring barley yields in central Norway in three sub-periods from 1946 to 1960, 1960 to 1980 and 1980 to 2008 accounted for 29, 43 and 78 % of total gain; whereas, the average on-farm yield increase over the total period was 70 % (Lillemo et al. 2010). A very low improvement in dry matter yield (0.3 % p.a.) due to new perennial and Italian rye grass varieties was observed in Belgian VCU trials over the period 1963–2007 (Chaves et al. 2009). For Finnish cereal crops, Peltonen-Sainio et al. (2009) found that plant breeding has successfully increased genetic yield potential without any indication of reduced rates of improvement in recent years. For national on-farm yields, however, they report that yield trends declined for all cereal crops except wheat. Ahlemeyer and Friedt (2011) analysed 90 German winter wheat varieties registered between 1966 and 2007, based on trials grown at five sites and in 3 years. The genetic improvement in grain yield was between 0.340 and 0.375 dt ha^−1^ year^−1^. They found no hints, that stagnation in on-farm yield is due to lack in genetic improvement of varieties. In an earlier study of Schuster (1997), data on 11 major crops from German national registration and regional state trials in the federal state of Hessen, conducted from 1952 until 1993, were analysed and compared with national on-farm yields. Genetic and agronomic yield trends were calculated concluding that yield increase is linear for most crops (Henceforth, if we use the term “agronomic trend” we imply all non-genetically caused factors, not only agronomic practices, but also impact of climate change, change in agricultural policy measures, etc.). Among cereals, winter wheat showed the largest genetic trend and winter rye the lowest in grain yield with 0.51 and 0.10 dt ha^−1^ year^−1^, respectively. Based on the yield level of 1952, the total yield increase due to genetic improvement of varieties over all 11 crops ranged from 10 % for winter rye to 63 % for oil seed rape, respectively. Generally, yields from trial results are higher than on-farm yields. For German cereal crops, Schuster (1997) found a difference in yield level of 20–22 % in the period 1952 until 1997, and Rijk et al. (2013) reported relatively small, but diverging differences found in the Netherlands due to very favourable Dutch growing conditions. However, a stagnation or even decline of on-farm yield was observed (Brisson et al. 2010; Oury et al. 2012; Lin and Huybers 2012; Peltonen-Sainio et al. 2009; Ahlemeyer and Friedt 2011) in the last three decades. Possible sources for levelling-off in yield gain are mentioned: extensification of agronomic practices, reduction of fertilizer and agrochemicals, and extension of cropping towards less favourable areas.

In this paper, we study the performance trends of varieties tested during the last 30 years from 12 major crops in Germany. We first describe the datasets analysed and methods applied. Special attention will be paid to dissecting genetic and non-genetic sources of trend. For cereal crops, trends of ageing effects will be investigated by comparing fungicide and non-fungicide-treated trial pairs. National on-farm yields are compared with trial yield and yield gap trends are estimated. Relative yield gaps at the beginning and the end of our study period are reported. The paper ends with a discussion by comparing yield trends in trials across crops and with national on-farm yields, looking at relative yield gaps and considering possible causes for biased trend estimates.

## Materials and methods

### Datasets

Data from 12 different crops of German official variety trials were analysed over the period from 1983 to 2012 (see Table 1). Besides the yield data some other important traits such as oil content of winter oilseed rape were included in our investigation. For cereals, two different intensities of treatment were applied. Intensity 2 comprises best local agronomic practice in fertilizer, fungicide and other agrochemical treatment. For intensity 1, no fungicides and growth regulators were applied; from 2005 on, the other treatments were applied at the same level as in intensity 2. Before 2005, for crops other than spring barley, nitrogen dressing was reduced by 30–40 kg/ha^-1^ N. Until 1992, varieties were tested at more than one level of fungicide treatment. In this case, intensities treated with different levels of fungicides were averaged and considered as intensity 2. For sugar beets, a second intensity of treatment was introduced with an additional fungicide application in 2001. However, only the untreated intensity was used for analysis in this paper. The “normal” trial series, grown on non-infected Rhizomania locations, was analysed. Since 1985, forage and grain maize varieties are arranged into maturity groups “early”, “medium” and “late”. For both crops, we analysed the medium group only, which is the largest one. Until 2002, all maize varieties had to be tested in the forage series. From 2003 on, testing in the forage series was no longer obligatory. From that time on, less grain type maize varieties were included in the forage series. In fodder grass trials, up to ten cuts per year were harvested in three successive years. The year of sowing was not counted as harvest year. Italian ryegrass is sown each year, whereas perennial ryegrass has three harvest years of the same trial, which can be considered as repeated measures. In order to deal with the correlation between harvest years of the same trial, only the averages of the 3 years were used for analysis. This approach has the advantage that the analysis of means across 3 years proceeds in the same way as the analysis of annual crops. As means from two adjacent series share 2 years of testing, the analysis can only be regarded as approximate. This partial overlap implies that the means across 3 years are not entirely independent (Piepho and Eckl 2014). We studied three variables: the sum over all cuts, the first cut and the sum of the second and consecutive cuts.

**Table 1:** Basic information on VCU performance trial data

Crop	Trait	No. of observations	Total no. of varieties	Average no. of varieties tested per year	Standard varieties			No. of years	No. of locations	Percentage of variety–year–location- combinations
					No. of varieties	First year in trial	Average no. of years in trial			
Winter wheat	Grain yield (dt ha^−1^)	22,820	286	33.5	37	1963	7.4	30	115	2.13
Winter barley (two-row)	Grain yield (dt ha^−1^)	11,801	129	17.3	29	1972	7.1	29	125	2.29
Winter barley (six-row)	Grain yield (dt ha^−1^)	11,424	127	16.7	34	1969	6.4	29	122	2.28
Winter rye	Grain yield (dt ha^−1^)	9,545	86	13.2	26	1955	7.7	28	100	3.40
Winter triticale	Grain yield (dt ha^−1^)	6,661	65	11.3	25	1987	7.0	25	81	4.39
Spring wheat	Grain yield (dt ha^−1^)	6,745	61	10.5	25	1961	7.6	29	97	3.33
Spring barley	Grain yield (dt ha^−1^)	15,871	176	22.2	37	1971	7.1	30	113	2.37
Grain Maize	Grain yield (dt ha^−1^)	10,237	196	20.2	37	1972	5.8	26	116	1.61
Forage Maize	Total fresh matter yield (dt ha^−1^)	12,218	232	23.8	35	1972	6.3	26	105	1.84
Forage Maize	Total dry matter yield (dt ha^−1^)	12,205	232	23.8	35	1972	6.3	26	105	1.83
Winter oil seed rape	Grain yield (dt ha^−1^)	7,897	154	19.2	48	1973	6.1	30	96	1.59
Winter oil seed rape	Oil yield (dt ha^−1^)	7,289	154	19.1	46	1973	6.1	30	94	1.59
Sugar beet	Root yield (dt ha^−1^)	9,329	224	22.8	38	1963	6.9	30	78	1.58
Sugar beet	Crude sugar yield (dt ha^−1^)	9,269	224	22.8	38	1963	6.9	30	78	1.57
Perennial ryegrass	Dry matter yield (dt ha^−1^)	4,933	254	16.4	74	1956	9.7	28	27	2.60
Italian ryegrass	Dry matter yield (dt ha^−1^)	4,134	88	11.9	31	1956	7.6	30	26	5.55

The trials of all crops were about equally distributed across the individual crop’s typical growing regions. Before 1990, only data from West German locations were used. For cereals, the trials were laid out in split-plot designs with main plots arranged in complete blocks. The treatments were applied to main plots, and the varieties were arranged in subplots. Subplots within main plots were either laid out as randomized blocks, or as *α*-lattice designs, if there were more than 40 entries in a trial. For the other crops, randomized complete block designs with three or four replications were applied. The trial period for new varieties was 3 years for cereals, winter oilseed rape and fodder grasses and 2 years for maize and sugar beets. For each crop, 8–25 trials per testing year were conducted. The plot size varies, depending on years and crops, from 7 to 30 m^2^ with an average of 10 m^2^. We analysed only varieties registered for their “value of cultivation and use (VCU)”. Varieties registered for export purposes only, e.g. sugar beets, were excluded from the analysis. For each crop and trial, at least three standards running in trials for several years were included. Well established varieties were chosen as standards representing the actual state of breeding progress.

Prior to this study, the data were checked for recording errors and outliers as part of the evaluation process for registering varieties for the German National List. Additionally, in order to avoid biased results, we checked data thoroughly for its consistent structure over time before we carried out analysis. Inconsistent data structures may have occurred due to changes in assessment of a characteristics’ scale of measurement or structure of trial series. We checked which level of intensity corresponds to intensity 2 (with fungicide treatment), if there were two or even three fungicide levels applied within a trial. In such cases, we dropped one or two levels and kept the appropriate one or just used the average, if appropriate. In other cases, as for sugar beets and maize, it has to be taken into account that the testing period has been shortened by 1 year. In order to find out such types of inconsistent data, we manually screened historic testing reports and trial plans up to 1991. In later years the necessary information was already stored with the trial data. The data analysis was based on means over replications per trial site. We obtained cropping area and average on-farm yield in Germany from the national census (DESTATIS, https://www-genesis.destatis.de) (Table 1).

### Statistical analysis

#### Model for genetic and non-genetic trend

For a given intensity, we used the standard three-way model with factors genotype, location and year given by Laidig et al. (2008)

1 $$y_{ijk} = \mu + G_{i} + L_{j} + Y_{k} + \left( {LY} \right)_{jk} + \left( {GL} \right)_{ij} + \left( {GY} \right)_{ik} + \left( {GLY} \right)_{ijk} ,$$

where *y*_*ijk*_ is the mean yield of the *i*th genotype in the *j*th location and *k*th year, *μ* is the overall mean, *G*_*i*_ is the main effect of the *i*th genotype, *L*_*j*_ is the main effect of the *j*th location, *Y*_*k*_ is the main effect of the *k*th year, (*LY*)_*jk*_ is the *jk*th location × year interaction effect, (*GL*)_*ij*_ is the *ij*th genotype × location interaction effect, (GY)_*ik*_ is the *ik*th genotype × year interaction effect, and (*GLY)*_*ijk*_ is a residual comprising both genotype × location × year interaction as well as the error of the mean over replications from a randomized block, split-plot or adjusted mean from a *α*-lattice design. This model assumes that locations are crossed with years, i.e. at least some locations are used across several years. All effects except *μ*, *G*_i_ and *Y*_*k*_ are assumed to be random and independent with constant variance for each effect. Genetic and non-genetic time trend were studied by modelling *G*_*i*_ and *Y*_*k*_ with regression terms for time trends as follows (Piepho et al. 2014):

2 $$G_{i} = \beta r_{i} + H_{i} ,$$

where *β* is a fixed regression coefficient for genetic trend, *r*_*i*_ is the first year of testing for the *i*th variety, and *H*_*i*_ models a random normal deviation of *G*_*i*_ from the genetic trend line, and

3 $$Y_{k} = \gamma t_{k} + Z_{k} ,$$

where *γ* is a fixed regression coefficient for the non-genetic trend, *t*_*k*_ is the continuous covariate for the calendar year and *Z*_*k*_ is a random normal residual. Genetic and non-genetic trends are quantified by the regression coefficients *β* and *γ*, respectively, indicating the yield increase per year measured in the same units as *y*_*ijk*_.

#### Model for overall trend

Overall trend was modelled considering the genotype as nested within years. Thus, compared with model (1), for this analysis we dropped effects involving genotypes that are not nested within years, i.e. we dropped the effects *G*_*i*_ and (*GL*)_*ij*_. The reduced model is given by

4 $$y_{ijk} = \mu + L_{j} + Y_{k} + \left( {LY} \right)_{jk} + \left( {GY} \right)_{ik} + \left( {GLY} \right)_{ijk}$$

Similarly as in Eq. (3), the year main effect can be modelled as

5 $$Y_{k} = \varphi t_{k} + U_{k} ,$$

where *φ* is a fixed regression coefficient for overall trend, *t*_*k*_ is the continuous covariate for the calendar year and *U*_*k*_ is a random residual following a normal distribution with zero mean and variance $\sigma_{U}^{2}$. We take the year main effects as fixed in order to obtain adjusted means for years, representing the overall trend.

#### Model for trend of average national on-farm yield

For estimating the trend of average national on-farm yield, we used the simple regression model

6 $$y_{k} = \mu + \omega t_{k} + e_{k} ,$$

where *ω* is a fixed regression coefficient for on-farm yield trend, *t*_*k*_ represents the calendar year and *y*_*k*_ the average on-farm yield in year *k*.

#### Model for trend of gap between trial means and on-farm yields

To find out whether there is a trend in the absolute gap between trial yield and on-farm yield, we calculated the regression of the difference between overall trial means and on-farm yields using model (6).

#### Relative magnitude of yield gap in 1983 and 2012

In order to quantify yield gaps at the beginning and at the end of studied period, we calculated the differences as the vertical distance between the linear regression of overall trial and on-farm yield 1983 and 2012 and expressed the differences as percentage of the overall regression at calendar years 1983 and 2012.

#### Effect of variety ageing

The fixed part of the trend model developed so far has two linear components using models (1), (2) and (3).

7 $$\eta_{ik} = \mu + \beta r_{i} + \gamma t_{k} ,$$

where *η*_*ik*_ is the expected response of the *i*th genotype in the *k*th year. If we assume that a linear age-dependent trend exists, then (7) extends to

8 $$\eta_{ik} = \mu + \beta r_{i} + \gamma t_{k} + \delta a_{ik} ,$$

where $a_{ik} = r_{i} - t_{k}$ is the age of variety *i* at testing year *k* and *δ* denotes the fixed ageing effect. Because of the linear dependence of *a*_*ik*_ on *r*_*i*_ and *t*_*k*_, the three regression parameters are not independently estimable. By rearranging Eq. (8) we get

9 $$\eta_{ik} = \mu + \tilde{\beta }r_{i} + \tilde{\gamma }t_{k} ,$$

where

10 $$\tilde{\beta } = \beta - \delta$$

and

11 $$\tilde{\gamma } = \gamma + \delta .$$

If there exists an ageing effect *δ* < 0, then we should keep in mind that the genetic trend $\tilde{\beta }$ has an upward and the non-genetic trend $\tilde{\gamma }$ a downward bias.

For cereal crops tested with two intensities, we investigated the influence of the variety age *a*_*ik*_ on variety performance by comparing intensity 2 and intensity 1. The treatment difference was calculated for each trial pair. If we assume that the regression parameters for the genetic and the non-genetic trend are the same for both intensities, then the fixed part of our regression model for the difference now can be written as

12 $$\eta_{ik 2} - \eta_{ik 1} = \mu_{ 2} - \mu_{ 1} + (\delta_{ 2} - \delta_{ 1} )a_{ik} ,$$

where the suffixes 1 and 2 denote the intensity of the effects by using the fixed parts of Eqs. 1, 2 and 3. We assume that genetic and non-genetic trends are equal under both intensities. *δ*_2_ < 0 and *δ*_1_ < 0 are the negative effects of variety age for intensity 2 and 1. Further let *δ*_*d*_ = *δ*_2_−*δ*_1_, then *δ*_*d*_ > 0 will be the trend of variety ageing for the difference of intensity 2 − intensity 1, assuming that *δ*_1_ < *δ*_2_. If fungicide treatment fully compensates for ageing (*δ*_2_ = 0), then the ageing effect of the difference is positive with *δ*_*d*_ =−*δ*_1_.

Applying model (1) to treatment differences, the trend of variety age can be incorporated into the genotype–year interaction effect, using the assumptions that genetic and non-genetic trends are the same under both treatments. We replace the genotype–year interaction by

13 $$(GY)_{ikd} = \delta_{d} a_{ik} + (ZH)_{ikd} ,$$

where *δ*_*d*_ is the fixed regression coefficient for the trend of variety ageing and (*ZH*)_*ikd*_ the random deviation of the interaction term from the linear trend.

#### Graphical displays

We define a fixed categorical effect *C*_*p*_ for groups *p* = 1*,…P*, where *P* is the number of levels of the time variable *r*_*i*_, where each group is represented by at least one genotype. Then, the genetic effect can be modelled as

14 $$G_{i} = C_{p} + H_{i} ,$$

where *H*_*i*_ is the random deviation from the trend, as given in (2). We compute adjusted means for *C*_*p*_ and plot them against first year of testing (*r*_*i*_). Similarly, the age of a variety may be modelled as

15 $$(GY )_{ikd} = D_{q} + (ZH )_{ikd}$$

where* D*_q_ (*q* = 1,….,*Q*) is a categorical effect for the *q*th age class and *Q* is the maximum age of a variety (Piepho et al. 2014).

The following plots can be considered to be based on the proposed models:

(1) Visible genetic trend: plot of adjusted genotype-group means for *C*_*p*_ based on (14), inserted in the baseline model (1), against time (*r*_*i*_).

(2) Visible agronomic trend: plot of adjusted year means for *Y*_*k*_ using the baseline model (1) against calendar year *t*_*k*_.

(3) Visible overall trend: plot of adjusted year means for *Y*_*k*_ of model (4)  against calendar year *t*_*k*_.

(4) Visible trend of average national on-farm yield: plot of annual average yield on calendar year of harvest.

(5) Visible age effect: plot of adjusted means for *D*_*q*_ against age (*a*_*ik*_) based on model (15).

## Results

**Fig. 1:**
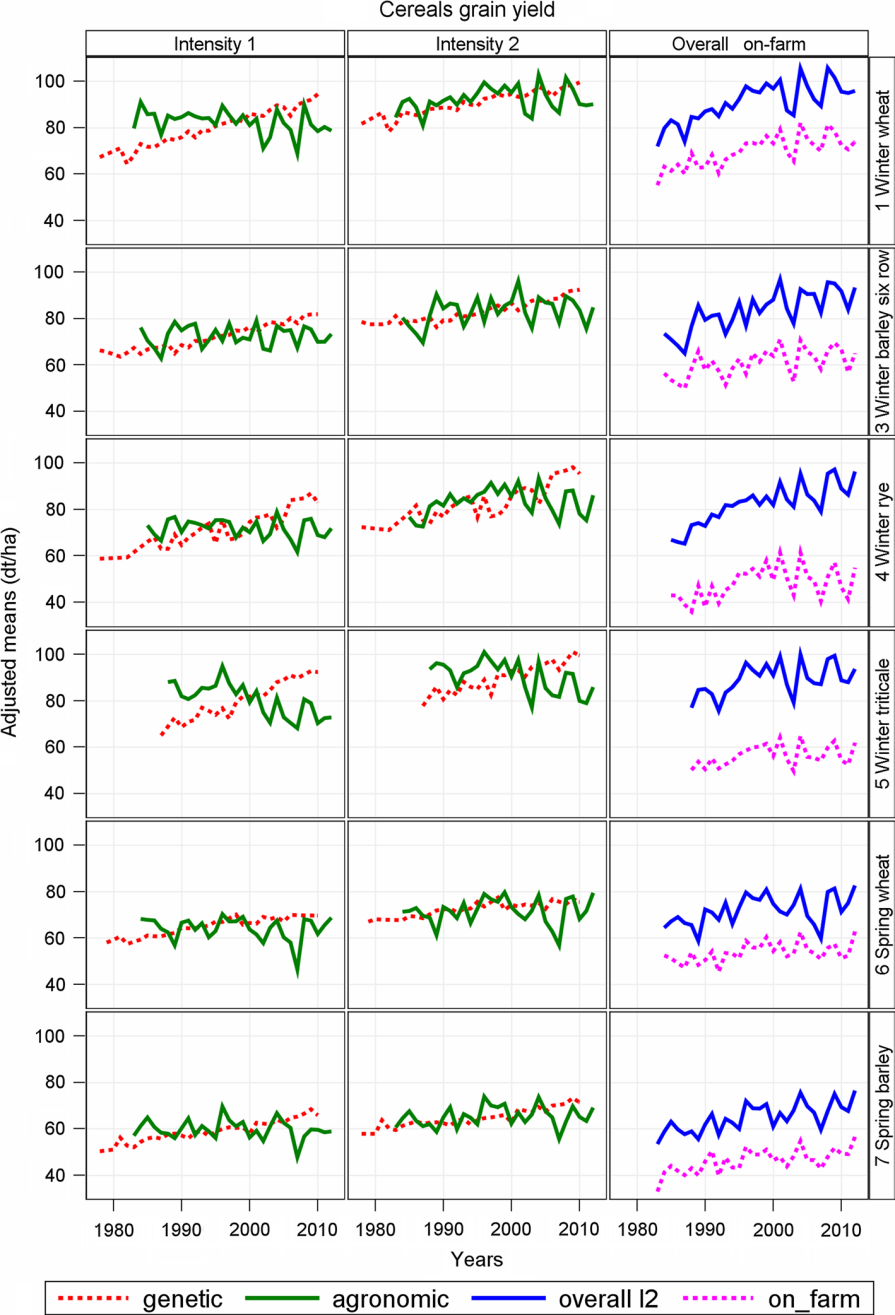
Adjusted yield means of winter wheat, six-row winter barley, winter rye, winter triticale, spring wheat and spring barley for intensity 1 and intensity 2. Genetic: variety group means (effect *C*_*p*_ in Eq. 14). Agronomic: year means (Eq. 1, using Eq. 14 to model* G*_*i *_analog). Overall: overall year means for intensity 1 (Eq. 4 using Eq. 14 to model *G*_*i*_ analog). On-farm: national average on-farm yields

In Tables 2, 4, 5 we compared the overall trend obtained in variety trials with the national average on-farm yield from 1983 to 2012. Generally, on-farm yields are lower than trial yields. However, it is of interest whether the gap stays constant over time (parallel lines) or if it is widening. If relative yield gaps 1982 and 2012 are of the same magnitude, then the gap is increasing proportionally. Therefore, we compared the trends of both series. Further, we dissected trends into a genetic and a non-genetic (agronomic) part to quantify the impact of plant breeding on the one hand and further trend influencing factors, which we denoted as agronomic, on the other hand. In order to compare trends across crops within this study, we expressed the regression estimates as percentage of the yield level in 1983, calculated from the linear overall and on-farm regression line, respectively. (Percentage figures between yield progress studies should be compared cautiously, because different baselines might have been used. Lobell et al. (2009) apply the most recent average potential yield as basis, whereas Fischer and Edmeades (2010) and Fischer et al. (2014) prefer the recent on-farm value.) The series plots for the agronomic, overall and on-farm means (Figs. 1, 3, 4) are apparently congruent in their pattern indicating that variation of yield response in trials is like those observed under farm conditions, i.e. trial yields and on-farm yields are highly correlated.

**Table 2:** Estimates of regression coefficients for performance trends in German official variety trials and in national on-farm harvests (Cereals, grain yield in dt ha^−1^)

Crop	Intensity	Baseline y (1983)		Estimates of linear yield trends															Yield gaps (%)	
				Genetic			Agronomic			Overall			On-farm			Gap overall—on-farm				
				Absolute	SE	%	Absolute	SE	%	Absolute	SE	%	Absolute	SE	%	Absolute	SE	%		
		Overall	On-farm																1983	2012
Winter wheat	I2	80.6	61.4	0.530^***^	0.042	0.66	0.161 ns	0.119	0.20	0.716^***^	0.123	0.89	0.567^***^	0.094	0.92	0.158^**^	0.056	0.20	24	23
	I1	70.8		0.817^***^	0.044	1.15	−0.197 ns	0.111	–0.28	0.651^***^	0.110	0.92								
Winter barley (two-row)	I2	68.1	54.5	0.558^***^	0.041	0.82	0.196 ns	0.121	0.25	0.768^***^	0.114	1.13	0.412^**^	0.106	0.76	0.379^***^	0.047	0.56	20	26
	I1	59.2		0.621^***^	0.038	1.05	0.012 ns	0.095	0.02	0.665^***^	0.088	1.12								
Winter barley (six-row)	I2	72.0	54.5	0.435^***^	0.043	0.60	0.289 ns	0.136	0.20	0.761^***^	0.129	1.06	0.412^**^	0.106	0.76	0.373^***^	0.041	0.52	24	29
	I1	62.1		0.534^***^	0.041	0.86	0.122 ns	0.110	–1.67	0.693^***^	0.101	1.12								
Winter rye	I2	68.9	41.9	0.656^***^	0.070	0.95	0.134 ns	0.141	0.19	0.867^***^	0.121	1.26	0.392^*^	0.130	0.93	0.525^***^	0.091	0.76	39	43
	I1	59.7		0.696^***^	0.061	1.17	−0.088 ns	0.105	–0.15	0.699^***^	0.106	1.17								
Winter triticale	I2	81.9	52.3	0.918^***^	0.083	1.13	−0.427 ns	0.188	–0.53	0.433^*^	0.183	0.58	0.235 ns	0.117	0.45	0.232^**^	0.078	0.28	36	37
	I1	68.9		1.178^***^	0.082	1.71	−0.534 ns	0.165	–0.77	0.567^***^	0.154	0.82								
Spring wheat	I2	66.8	49.2	0.328^***^	0.040	0.49	0.035 ns	0.139	0.05	0.336^*^	0.138	0.50	0.288^**^	0.087	0.58	0.116 ns	0.096	0.17	26	25
	I1	60.0		0.403^***^	0.034	0.67	−0.103 ns	0.124	–0.17	0.293^*^	0.121	0.49								
Spring barley	I2	57.9	40.6	0.391^***^	0.036	0.68	0.093 ns	0.098	0.16	0.483^***^	0.096	0.84	0.379^***^	0.080	0.93	0.114^*^	0.040	0.20	30	28
	I1	53.8		0.455^***^	0.036	0.85	−0.081 ns	0.095	–0.15	0.374^***^	0.093	0.70								

Percent trends (%) are based on 1983 performance levels. Yield gaps at baselines 1983 and 2012 as percent of linear overall regression estimates

*ns* not significant at 5 % level, *SE* standard error

* Significant at 5 % level

** Significant at 1 % level

*** Significant at 0.1 % level

**Table 3:** Estimates of regression coefficients of variety ageing in cereal crops for grain yield as difference of intensity 2 and intensity 1 in German official variety trials

Crop	Estimates of regression coefficients for ageing *δ* ^*d*^		
	Absolute	SE	%
Winter wheat	0.287^***^	0.024	0.36
Winter barley (two-row)	0.070^**^	0.026	0.10
Winter barley (six-row)	0.109^***^	0.026	0.15
Winter rye	0.043 ns	0.022	0.06
Winter triticale	0.316^***^	0.057	0.39
Spring wheat	0.092^***^	0.022	0.14
Spring barley	0.067^***^	0.014	0.12

Percent trends (%) are based on 1983 performance levels

*ns* not significant at 5 % level, *SE* standard error

*%* Regression coefficient as percent of baseline overall 1983

* significant at 5 % level

** significant at 1 % level

*** significant at 0.1 % level

**Table 4:** Estimates of regression coefficients for performance trends in German official variety trials and in national on-farm yields (Maize, Sugar beet, Rape)

Crop	Trait	Baseline y (1983)		Estimates of linear yield trends															Yield gaps (%)	
				Genetic			Agronomic			Overall			On-farm			Gap overall—on-farm				
				Absolute	SE	%	Absolute	SE	%	Absolute	SE	%	Absolute	SE	%	Absolute	SE	%		
		Overall	On-farm																1983	2012
Grain Maize	Grain yield (dt ha^−1^)	87.9	61.9	1.587^***^	0.066	1.80	−0.305 ns	0.200	−0.35	1.559^***^	0.189	1.77	1.329^***^	0.125	2.24	0.243 ns	0.118	0.28	30	24
Forage Maize	Total dry matter (dt ha^−1^)	170.8		1.917^***^	0.107	1.12	−0.650^*^	0.325	−0.38	1.409^***^	0.314	0.82								
Forage Maize	Total fresh matter (dt ha^−1^)	549.9	438.0	6.424^***^	0.503	1.14	−4.249^***^	1.220	−0.75	2.455^*^	1.177	0.45	−0.044 ns	0.682	−0.01	2.136^**^	0.760	0.39	20	30
Winter oil seed rape	Grain yield (dt ha^−1^)	37.1	27.8	0.528^***^	0.028	1.42	0.019 ns	0.080	0.05	0.543^***^	0.076	1.46	0.342^***^	0.077	1.23	0.204^**^	0.073	0.55	25	29
Winter oil seed rape	Oil yield (dt ha^−1^)	14.6		0.272^***^	0.014	1.86	0.018 ns	0.037	0.12	0.292^***^	0.035	2.00								
Sugar beet	Root yield (dt ha^−1^)	600.6	448.7	3.593^***^	0.385	0.60	6.270^***^	1.023	1.04	10.458^***^	1.068	1.74	7.414^***^	0.816	1.65	2.805^**^	0.782	0.47	25	27
Sugar beet	Crude sugar yield (dt ha^−1^)	105.3	75.0	0.768^***^	0.068	0.73	1.020^***^	0.213	0.97	1.879^***^	0.219	1.79	1.516^***^	0.155	2.02	0.331^*^	0.158	0.31	29	26
Sugar beet	Sugar yield (corr.) (dt ha^−1^)	90.2	64.9	0.747^***^	0.063	0.83	1.011^***^	0.196	1.12	1.841^***^	0.199	2.04	1.442^***^	0.140	2.22	0.376^**^	0.154	0.42	28	26
Sugar beet	Standard mollasses loss (%)	1.7		–0.007^***^	0.001	–0.38	–0.012^***^	0.002	–0.68	–0.185^***^	0.015	–1.08								

Percent trends (%) are based on 1983 performance levels. Yield gaps at baselines 1983 and 2012 as per cent of linear overall regression estimates

*ns* not significant at 5 % level, *SE* standard error

* Significant at 5 % level

** Significant at 1 % level

*** Significant at 0.1 % level

**Table 5:** Estimates of regression coefficients for performance trends in German official variety trials (Ryegrass).

Crop	Trait	Baseline y (1983) Overall	Estimates of linear yield trends								
			Genetic			Agronomic			Overall		
			Absolute	SE	%	Absolute	SE	%	Absolute	SE	%
Perennial ryegrass	Total dry matter yield (dt ha^−1^)	120.11	0.453^***^	0.029	0.38	−0.344^*^	0.147	−0.29	0.174 ns	0.151	0.15
Perennial ryegrass	Dry matter yield cut 1 (dt ha^−1^)	57.32	0.264^***^	0.042	0.46	−0.404^***^	0.102	−0.71	–0.104 ns	0.109	–0.18
Perennial ryegrass	Dry matter yield sum cut 2 to n (dt ha^−1^)	62.54	0.195^***^	0.025	0.31	0.075 ns	0.099	0.12	0.297^**^	0.098	0.47
Italian ryegrass	Total dry matter yield (dt ha^−1^)	172.33	0.271^***^	0.036	0.16	0.214 ns	0.296	0.12	0.508 ns	0.302	0.29
Italian ryegrass	Dry matter yield cut 1 (dt ha^−1^)	69.20	0.016 ns	0.020	0.02	−0.516^**^	0.181	−0.75	−0.492^**^	0.184	−0.71
Italian ryegrass	Dry matter yield sum cut 2 to n (dt ha^−1^)	102.95	0.262^***^	0.037	0.25	0.751^**^	0.230	0.73	1.037^***^	0.238	1.01

Percent trends (%) are based on 1983 performance level

*ns* not significant at 5 % level, *SE* standard error

* Significant at 5 % level

** Significant at 1 % level

*** Significant at 0.1 % level

### Cereals (Table 2; Fig. 1)

In the fungicide-treated series, winter wheat, winter barley and winter rye show overall yield trends (for intensity 2) above 0.7 dt ha^−1^ year^−1^, whereas trend rates for winter triticale, spring wheat and spring barley are below 0.5 dt ha^−1^ year^−1^ (Table 2). When looking at the percent overall trends (based on the crops’ 1983 yield level), it can be seen that spring wheat and winter triticale trends are less than 0.6 % p.a., whereas for winter barley and winter rye the annual increase in yield performance exceeds 1 % p.a. (Table 2). For all cereal crops, the trend in yield gap increases significantly in the range of 0.17–0.76 % p.a. (Table 2). A considerable discrepancy between trial and on-farm trend exists for winter rye (0.525 dt ha^−1^ year^−1^) and winter barley (0.373 dt ha^−1^ year^−1^) (Table 2). For these two crops, trial yields gain almost twice as much over the last 30 years than farm harvests. As expected, overall trends and yield levels for the intensity 1, which received no fungicide treatment, are substantially lower for all cereals with only one exception: for winter triticale the yield gain in intensity 1 (0.567 dt ha^−1^ year^−1^) is considerably higher than for intensity 2 (0.433 dt ha^−1^ year^−1^(Table 2).

We compared the regression estimates for genetic and agronomic trends and found that for all cereals a much larger increase in yield is caused by new varieties than by agronomic factors. Due to the large variability of the year responses (Fig. 1), the estimates of the agronomic trends turned out to be not significant (Table 2). It seems that genetic progress in yield was nearly linear over the last 30 years (Fig. 1). Table 2 indicates that for all cereal crops yield gaps 2012 were in the range of 23–43 %.

### Effect of ageing in cereals (Table 3; Fig. 2)

**Fig. 2:**
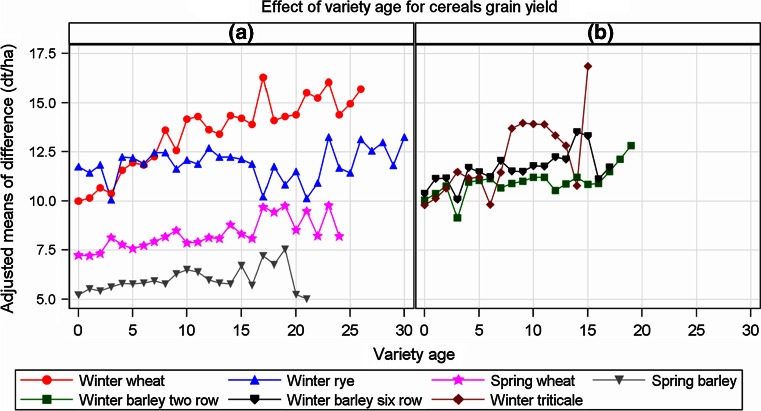
Effect of variety age on difference between intensity 2 and intensity 1 for grain yield of cereal crops [Model (1) combined with Eq. 13]

The summary of the regression results for agronomic trends in Table 2 shows lower rates for intensity 1 than for intensity 2 and most of them are even negative. However, higher rates for genetic trends are found. We assume that this pattern is connected with a loss of performance due to ageing effects of varieties in intensity 1. In order to investigate the ageing effect, we analysed the yield difference as described in “Materials and methods” (Eqs. 12, 13). Figure 2 plots the adjusted means against the variety age, which is the difference between the actual testing year and the first year in test. The summary of regression coefficients for ageing effects in Table 3 indicates a strong decline of yield for winter wheat of 0.287 dt ha^−1^ year^−1^(winter triticale with 0.316 dt ha^−1^ year^−1^ is greatly influenced by year 15 and should be discounted because this point is based on a single variety, Fig. 2b), whereas winter barley, spring wheat and spring barley effects decrease moderately in the range of 0.067 and 0.109 dt ha^−1^ year^−1^. For winter rye, a rather slight non-significant decline of 0.043 dt ha^−1^ year^−1^ was observed.

### Maize, winter oilseed rape and sugar beets (Table 4; Fig. 3)

**Fig. 3:**
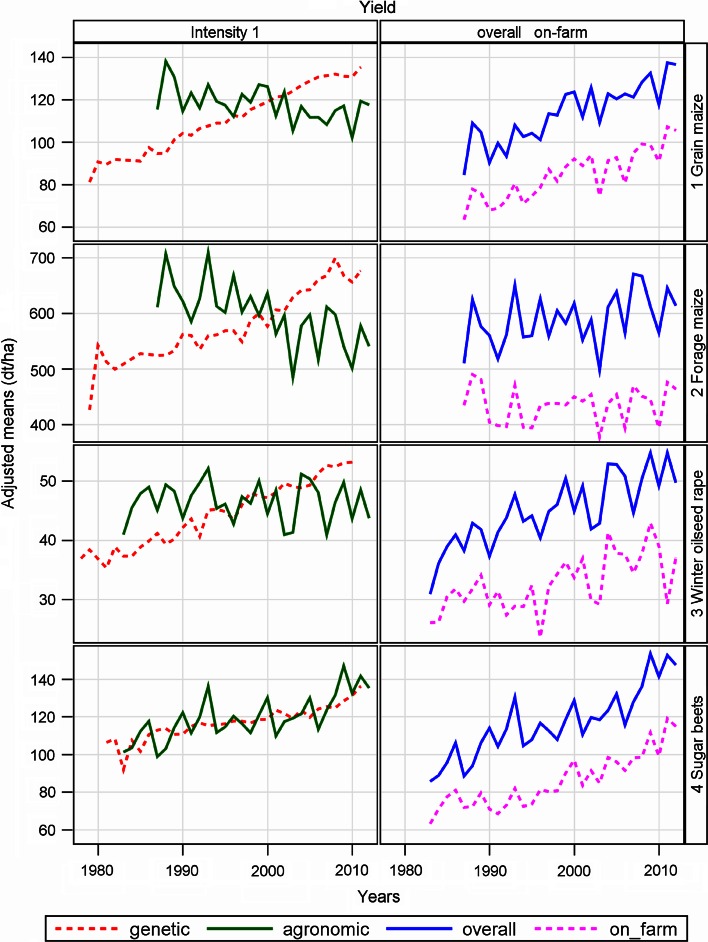
Adjusted yield means of grain maize (grain yield), forage maize (fresh matter yield), winter oilseed rape (grain yield), sugar beets (trial: corrected sugar yield, on-farm: white sugar yield). Intensity 1: only one treatment. Genetic: variety group means (effect *C*_*p*_ in Eq. 14). Agronomic: year means [Eq. (1), using Eq. (14) to model *G*_*i*_ analog]. Overall: overall year means for intensity 1 (Eq. 4 using Eq. 14 to model *G*_*i*_ analog). On-farm: national average on-farm yields

In this group, we obtained very different results across species with respect to yield levels, genetic and agronomic trends (Fig. 3). The most remarkable differences were observed in maize. In grain yield high gains were obtained for genetic (1.80 % p.a.), overall (1.77 % p.a.) and on-farm trend (2.24 % p.a.). In fresh matter yield the corresponding figures were 1.14, 0.45 and −0.01 % p.a., respectively (Table 4). In fresh matter yield, we found a significant increase in gap trend of 0.39 % p.a., whereas that for grain of 0.28 % was not significant. Comparison of the relative gaps highlight the difference between grain and forage: it shrinks from 30 to 24 % in grain and widens from 20 to 30 % for forage from 1983 to 2012 (Table 4). Compared with other crops in this group, considerable negative agronomic trends are found in maize.

For winter oilseed rape, the overall trend of 0.292 dt ha^−1^ year^−1^ (2.00 % p.a.) for oil yield was nearly completely due to genetic improvement (Table 4; Fig. 3).

For sugar beets, except root yield, we analysed the important trial traits crude and corrected sugar yield and standard molasses loss. Corrected sugar yield is derived from crude sugar yield by subtracting standard molasses loss. Standard molasses loss is calculated from the potassium, sodium and *α*-amino nitrogen content as an indicator of the technical quality of sugar beet (Loel et al. 2014). The corresponding trait for on-farm yield is white sugar yield. White sugar yield is that amount of sugar, which is produced from the root yield of one hectare and it is comparable to the corrected sugar yield assessed in variety trials. In contrast to all other species in this group, only for sugar beets, the agronomic trend exceeds the genetic one markedly for all traits observed.

### Fodder grasses (Table 5; Fig. 4)

We were not able to compare trial results with on-farm data, because suitable on-farm yield data were not available for fodder grasses. For multi-cut fodder crops, it is desired to get about equal yields for all cuts. For that reason, we considered not only the total dry matter yield, but also the yields of cut 1 and the sum of cut 2 and consecutive ones as two additional traits.

**Fig. 4:**
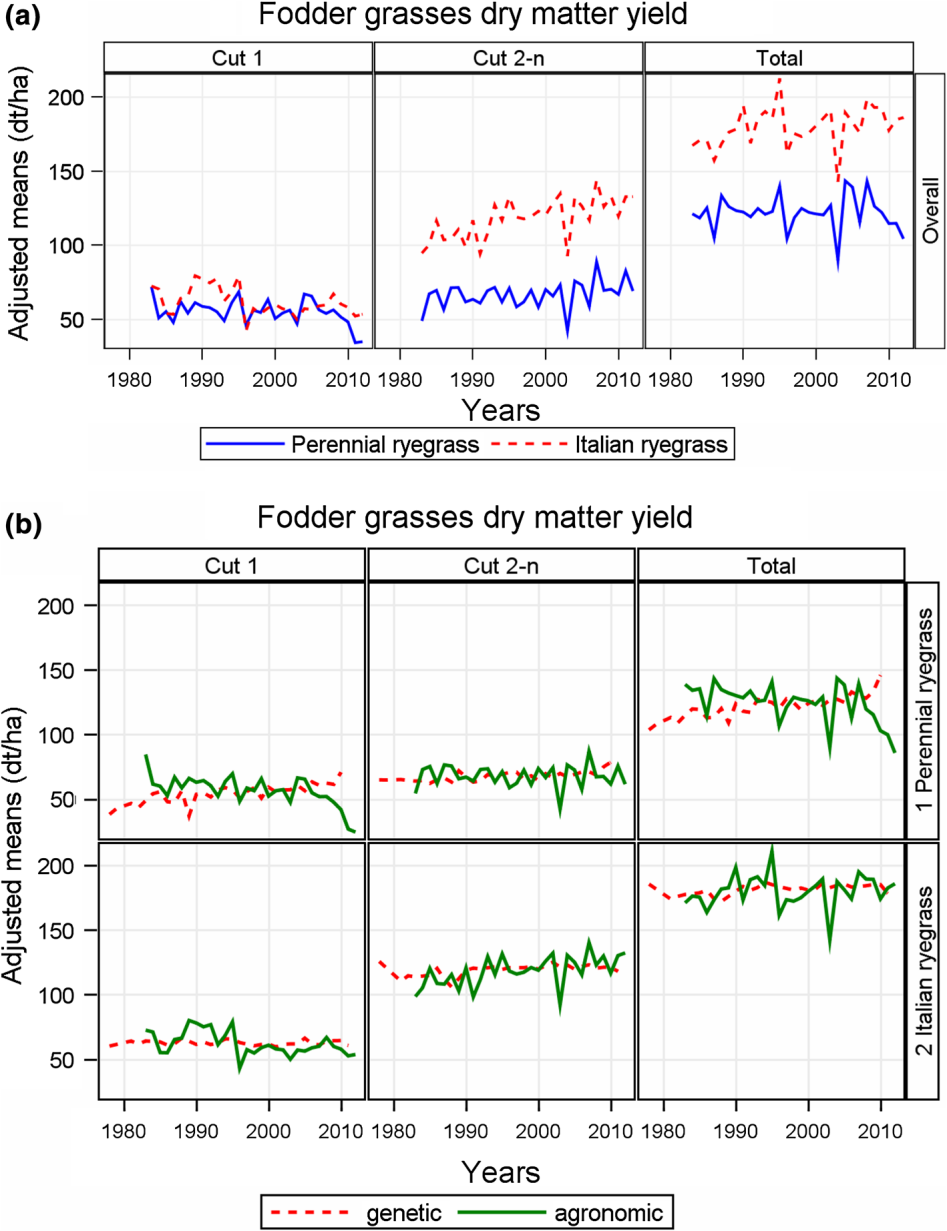
Adjusted dry matter yield means of perennial and Italian ryegrass. Cut 1: first cut, Cut 2-n: sum of second and consecutive cuts. Total: sum of all cuts. **a** Overall trends. **b** Genetic and agronomic trends. Genetic: variety group means (effect *C*_*p*_ in Eq. 14). Agronomic: year means [Eq. (1), using Eq. (14) to model *G*_*i*_ analog]. Overall: overall year means [(Eq. (4), using Eq. (14) to model *G*_*i*_ analog]

We first look at the overall trends in Fig. 4a which show that the total yield level of Italian ryegrass is about 50 % higher than that for perennial ryegrass. However, this is not true for the first cut, where yield levels for both ryegrasses are about the same. For perennial ryegrass, the gain in overall trend of total yield was only 0.174 dt ha^−1^ year^−1^, whereas that for Italian ryegrass it was 0.508 dt ha^−1^ year^−1^. For both crops the gain in dry matter yield was generated by the sum of cut 2 and higher (Table 5; Fig. 4a). For both crops, the percentage rates of overall yield indicate only a moderate, yet not significant gain, namely 0.15 % p.a. for perennial ryegrass and 0.29 % p.a. for Italian ryegrass (Table 5).

When looking at genetic and agronomic trends, Fig. 4 b and Table 5 show that for total yield, genetic trends for both grasses are significantly positive with 0.453 and 0.271 dt ha^−1^ year^−1^, respectively, whereas the agronomic trend for perennial ryegrass is negative (−0.344 dt ha^−1^ year^−1^) and for Italian ryegrass positive (0.214 dt ha^−1^ year^−1^). For Italian ryegrass, cut 2 and higher, a remarkable high agronomic trend of 0.73 % was observed. In summary, overall percentage trends of the sum of cut 2 and consecutive ones for perennial (0.47 % p.a.) and Italian ryegrass (1.01 % p.a.) suggest that the observed slight yield progress in grasses during the last 30 years was mainly achieved due to rising yields in later cuts.

## Discussion

### Genetic, agronomic and overall trends in variety trials

Our model allows estimation of linear genetic and agronomic trends in one step, including all main and interaction effects by taking into account all variation, which usually exists in multi-environmental trials. The pure agronomic trend is estimated mainly from the data of the standard varieties running in trials for a longer period. If we speak about genetic trend, we should keep in mind that estimated genetic trend addresses pure genetic trend plus the part arising from interactions of new varieties with progress in management technology. In this study, the average time a standard variety stayed in trial was in the range of 6.1–9.7 years depending on the crop (see Table 1). Standard varieties are included in registration trials if they represent the state of breeding progress and if they are widely used in commercial farming. Newly registered varieties are continuously included into the block of standard varieties and older ones are dropped. This continued update of standards provides the link to varieties grown on-farm.

The agronomic trend shows large year-to-year variation and it is influenced by many confounded factors. Besides changes in management practice, climate change is very important in the development of crop yield and food security. In chapter 10 of Fischer et al. (2014), a thorough and global account on climate change and its impact on crop yield is given. Results from studies on the influence of increase of growing season temperature to on-farm crop yields of the last 20–30 years are: for German wheat a decrease of −1.6 %, for France and the USA of −3.0 % and −3.2 % per 1 °C increase in temperatures; for northern Europe spring wheat yield loss ranges from −0.6 to −1.3 %. Oury et al. (2012) found that for French bread wheat climatic factors constituted the main explanation for yield degradation from 2000 on. However, Rijk et al. (2013) found a continued linear increase of yield and a positive impact of climate change during the last 30 years. They concluded that the recent enormous progress achieved in Dutch on-farm yield of cereals, sugar beets and potatoes may, in addition to high-yielding varieties, be attributed to crop management and improvements from climate change. A positive impact of global warming on the level of forage production in grassland in France is expected (Durand et al. 2010). Simulation results predicted a yield increase of 5–20 % until year 2100 due to a longer vegetation period. Lanning et al. (2010) found from a hard red winter wheat study in US northern Great Plains during 1950–2007 that earlier planting due to warmer spring temperatures has helped to alleviate negative effects of higher temperatures during grain filling period. The mentioned examples indicate that climate change may have positive and negative impact on the agronomic trends, depending on crops, management practices and geographical conditions.

For all crops, the genetic trends show a significant linear increase in yield over the last 30 years. We found no indication that progress has been slowing down in recent years, which is in line with results found in other studies (e.g. Peltonen-Sainio et al. 2009; Ahlemeyer and Friedt 2011; Mackay et al. 2011; Brisson et al. 2010; Rijk et al. 2013). Among all crops investigated in this study, especially forage grasses show a rather low progress in yield. The lowest rate was found for Italian ryegrass total dry matter with a trend of 0.16 % p.a. The highest rate of genetic trends (1.86 % p. a) was found for oil yield of winter oilseed rape. This is more than ten times the rate observed for Italian ryegrass. This wide spread of genetic progress observed in our study may be caused by various factors. Among others, breeding technique, intensity of breeding activities and agronomic conditions of a crop’s growing regions due to a shift in acreage may have an impact. Figures 1, 3 and 4 show that the agronomic trend is overlaid by a large year-to-year variability with mostly non-significant regression coefficients for cereal crops. The percentage rates for agronomic trends vary between 1.12 % p.a. for sugar yield (corrected) and −0.75 % p.a. for forage maize fresh matter yield, excluding results for cereals grown under intensity 1. For maize yield, we observed rather high rates of genetic trend but also a markedly significant negative agronomic trend. Sugar beet is the only crop where the agronomic trend significantly exceeds its genetic in magnitude. Similar results are reported for UK (Mackay et al. 2011) and Dutch (Rijk et al. 2013) sugar beet trials. In the UK and Dutch studies, longer growing seasons due to earlier sowing and later harvest dates, better quality in growing advice by sugar companies, and shrinkage of total sugar beet acreage resulting in a loss of less fertile fields are mentioned as reasons.

For perennial ryegrass, we observed a genetic trend of 0.46 % p.a. for the first cut. This is a rather high rate, when compared with the trend in first cut of Italian ryegrass of 0.02 % p.a. This difference can partially be explained with a shift over time from early diploid to later tetraploid maturing varieties, which give higher yields in their first cut. The results for genetic and agronomic trend for perennial ryegrass total yield (0.38 % and −0.29 %) and Italian ryegrass (0.16 and 0.12 %) as given in Table 5 are in line with results from Belgian VCU trial (Chaves et al., 2009) observed between 1963 and 2007 (0.31 % due to new varieties averaged over both crops, however, no agronomic progress). The noticeable negative overall trends for the first cut and the relatively large trends for the sum of the later cuts may have likely been due to two reasons. Higher persistence of new varieties (Chaves et al. 2009) and longer vegetation periods (Durand et al. 2010) will increase yields at later cuts, whereas dry spring seasons lower first-cut yields.

Our overall yield trend estimates describe the annual yield progress in official VCU trials based on varieties to become registered and on already registered standard varieties grown under best local agronomic practice. The progress observed in trials varies in a remarkably wide range between 0.15 % p.a. for total dry matter yield of perennial ryegrass and 2.04 % p.a. for corrected sugar yield (see Tables 2, 4, 5). The overall trend pattern follows closely the agronomic trend pattern with regard to the year-to-year variation, because genetic progress is continuously increasing with relative small year-to-year variability (see Figs. 1, 3, 4).

### Variety ageing

Decreasing yielding ability of a variety with increasing age is a well-known effect, mainly caused by a gradual breakdown of its resistance to disease (Silvey 1986; Evans and Fischer 1999; Fischer and Edmeades 2010; Mackay et al. 2011; Piepho et al. 2014). We estimated this ageing effect by comparing treated with untreated trial pairs (Eqs. 12, 13).

Plots in Fig. 2 and regression coefficients in Table 3 indicate that considerable ageing effects exist for cereal crops, which received no fungicide treatment. The average decline of yield is between 0.043 and 0.316 dt ha^−1^ year^−1^ under the assumption that the fungicide-treated trials are not subject to ageing (*δ*_2_ = 0, refer to Eq. 12). This assumption may not hold generally, because there is evidence from trial results that fungicide control may not be fully effective. In the presence of age effects, genetic trends are biased upwards and the agronomic trends downwards, as Piepho et al. (2014) and Mackay et al. (2011) demonstrated. Genetic and agronomic trends are confounded with ageing effects which cannot be estimated separately, if there is only a single treatment available. Piepho et al. (2014) showed that the genetic trend under ageing can be expressed as $\tilde{\beta } = \beta - \delta$ (Eq. 10) and the agronomic trend as $\tilde{\gamma } = \gamma + \delta$ (Eq. 11), where *β* and *γ* are the unbiased (purely) genetic and agronomic trends and *δ* is the ageing effect, introducing the bias (Eqs. 9–11). This fundamental result needs to be considered carefully when interpreting trend estimates from historical trials. Confounding with ageing effect explains the observed higher genetic and lower agronomic trends for intensity 1 as compared with the rates of intensity 2. Evans and Fischer (1999) pointed out: “The apparent progress without fungicide was much greater than the true progress in yield potential revealed with it. The difference highlights the importance of “maintenance breeding”, and is a reminder of the likely cost to neglecting it”. “Maintenance breeding” can be considered as the sum of breeding activities after registration in order to maintain the genotypic identity of a variety according to seed legislation requirements. To find the unbiased trends (assuming *δ*_2_ = 0), for example for winter wheat intensity 1, we subtract the age effect of *δ*_*d*_ = 0.287 dt ha^−1^ year^−1^ (Table 3) from the genetic and adding the same figure to the agronomic trend (from Table 2). Then we get 0.530 dt ha^−1^ year^−1^ for the unbiased genetic and 0.090 dt ha^−1^ year^−1^ for the agronomic trend. The corrected estimates are closer to the corresponding estimates for genetic (0.530 dt ha^−1^ year^−1^) and agronomic trend rates (0.161 dt ha^−1^ year^−1^) of intensity 2 (Table 2).

However, as mentioned before, we are not able to prove that no ageing effects exist under intensity 2. There are good reasons not to neglect the possibility that moderate effects of variety age may as well be present in a fungicide-treated trial. Results from fungicide-treated variety trials, however, give evidence that disease infection still occurs at a lower level. The results further show, that fungicide application does not fully control disease, but has a certain buffering effect. Therefore, we should be aware that genetic progress of treated trials can be overestimated due to ageing effects. This is in line with results from estimates of genetic progress obtained by growing historical varieties in recent trials along with new ones. For treated winter wheat trials, Ahlemeyer and Friedt (2011) found a genetic trend of 0.34 dt ha^−1^ year^−1^, which is considerably lower than our result of 0.530 dt ha^−1^ year^−1^ (Table 2). Piepho et al. (2014) considered results of this study as reflecting the pure genetic trend. In a similar study of Ahlemeyer et al. (2008) for winter barley varieties released between 1959 and 2003, a genetic gain for six-row barley of 0.432 dt ha^−1^ year^−1^ (0.435 dt ha^−1^ year^−1^, our result in Table 2) and for the two-row types of 0.391 dt ha^−1^ year^−1^ (0.558 dt ha^−1^ year^−1^, our result in Table 2) was found. The result for two-rowed barley is in line with what we found for winter wheat, whereas for the six-rowed barely the ageing effect (*δ*_2_) seems to be small. We should be aware that results obtained from trials with historical varieties may also show biased trends due to neighbour effects, including shading by older, taller varieties or inappropriate planting densities (Evans and Fischer 1999).

For maize yield, we believe that the rather high rate for genetic trend is biased upwards and the strong negative agronomic trend biased downwards due to non-estimable ageing effects. In maize trials fungicide treatment is not controlled. However, the effect of yield reducing leaf diseases should not be overlooked. From Polish studies between 1976 and 1992 (Lisowicz 1995) a yield loss of 3.3 % p.a. was reported. A recent Danish study (Nistrup 2011) reports on increasing problems with leaf diseases in Europe due to a rapid growth of maize acreage. In the Danish study the comparison of fungicide treated vs untreated trials showed considerable yield losses. These results are in line with fungicide control trials with grain maize in Germany in 2010 and 2011, where yield losses up to 30 % were found (Urban et al. 2012). Our results from untreated cereal trials indicate that ageing effects exist. Hence, we believe that for maize the genetic trend is overestimated and the agronomic trend biased downwards. If we assume an ageing effect of the magnitude as for winter wheat of 0.36 % p.a. (Table 3) and then correct genetic and agronomic trends, we would obtain conservative estimates of 1.80–0.36 % = 1.44 % p.a. and −0.35 + 0.36 % = 0.01 % p.a. for genetic and agronomic trends in grain maize, respectively. Analogously, for forage maize the corrected genetic trend would reduce to 0.76 % p.a. and the agronomic to −0.02 % p.a. for fresh matter yield.

**Fig. 5:**
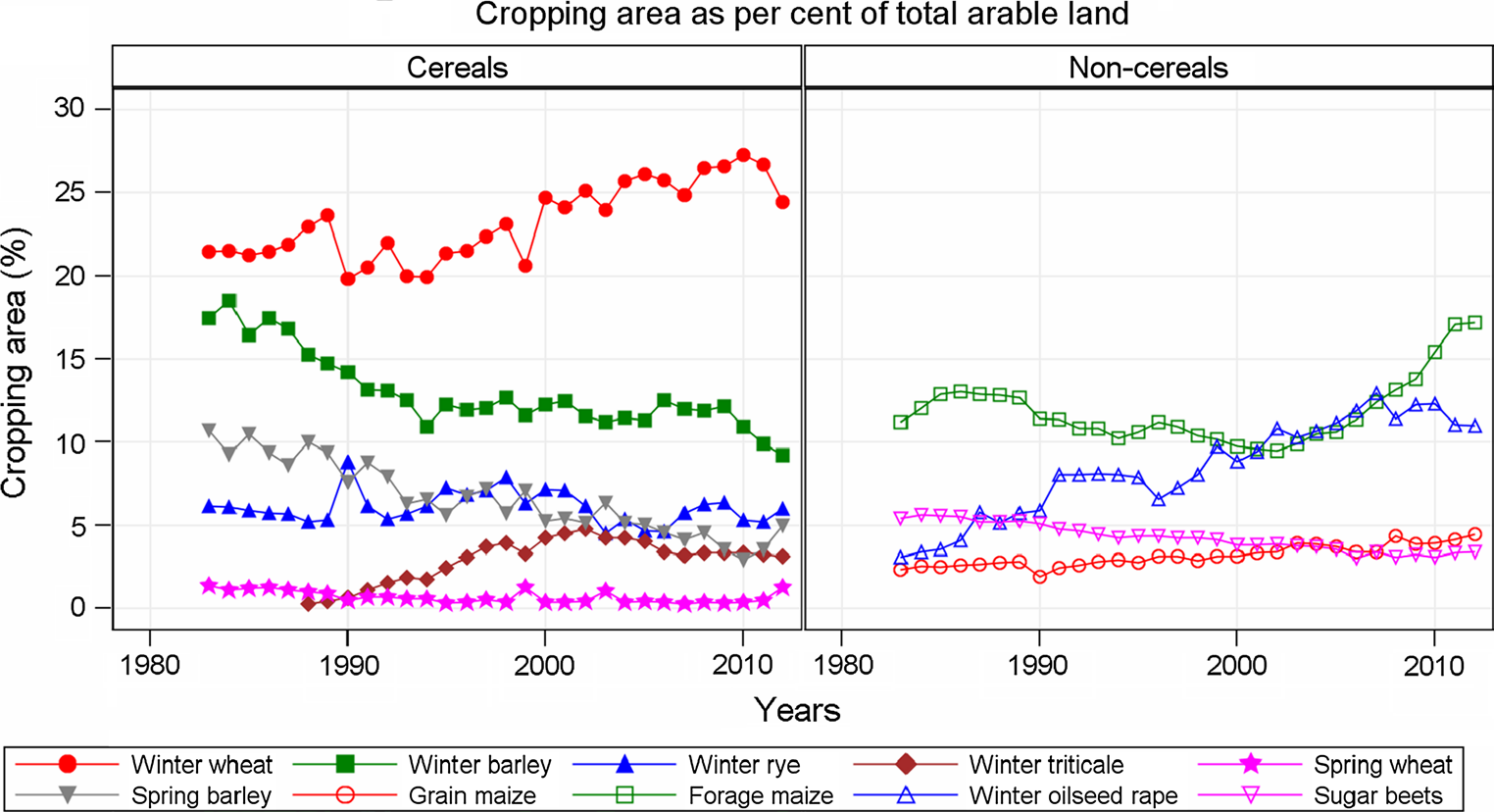
National growing area of major field crops as percentage of national arable land. Up to 1989, figures refer to West Germany only. Arable land in 1989 (West Germany): 7,272,701 ha, in 2012 (Germany): 11,850,000 ha

Further we should point out that winter oilseed varieties are tested without fungicide treatment. In recent years, however, leaf diseases have increased in this crop as a result of considerable extension of acreage (see Fig. 5). For this crop, we should assume biased trend effects due to variety ageing as well.

There may be another effect operating in variety trials leading to overestimation of genetic trends due to “regression to the mean” (Volker Michel, Landesforschungsanstalt für Landwirtschaft und Fischerei, Mecklenburg-Vorpommern, personal communication, 2014). In the context of plant breeding “Regression to the mean” is the phenomenon that if a certain fraction of the top yielding genotypes is selected, this fraction will tend to be closer to the mean in the next growing cycle. This phenomenon was first observed by Galton (1886). Our analysis is restricted to those varieties with at least 2 or 3 years of testing. Each year (seemingly) poor performers are discarded and a fraction of (seemingly) better performers are retained. But that selection is based on phenotypic data, not genotypic values. So in the following years there may be a “regression to the mean” in operation, which in turn may lead to a bias in our trend estimates. In order to explore this effect, we successively eliminated testing years from our data sets and then calculated genetic and agronomic trends. The results for winter wheat intensity 2 show a continuous decline of genetic and an increase of agronomic trends when eliminating the first (0.524, 0.171), first and second (0.489, 0.212) and then the first, second and third testing year (genetic trend: 0.469 dt ha^−1^ year^−1^, agronomic trend: 0.234 dt ha^−1^ year^−1^). Comparison with our results from the original data of 0.530 and 0.161 dt ha^−1^ year^−1^ for the genetic and agronomic trend, respectively (see Table 2), indicate a slight bias. Results for winter wheat intensity 1, spring wheat, fodder maize and winter oilseed rape are in line with winter wheat intensity 2 (data not shown).

Theoretical results on missing-data mechanisms suggest that all data should be used including varieties with just 1, 2 or 3 years of testing (Piepho and Möhring 2006). In order to investigate this, we estimated trends from winter wheat, forage maize and winter oilseed rape data including varieties which have been discarded after the first, second and third year (data not shown). However, the trend rates found were of about the same magnitude as our figures in Table 2, indicating that inclusion of non-registered varieties does not avoid bias.

These results suggest that bias may be introduced in trend estimates not only by ageing due to loss of disease resistance, but also from “regression to mean” effect if data are obtained under selection.

### Trial yield vs. national average on-farm yield

Recent studies have investigated the question if and why on-farm yields, mainly for winter wheat, are stagnating and if there is a gap between trial-station and national average on-farm yields (e.g. Lobell et al. 2009; Brisson et al. 2010; Fischer and Edmeades 2010; Oury et al. 2012; Fischer et al. 2014). Lin and Huybers (2012) estimated a change point from a linear increase to a levelling-off for German winter wheat on-farm yield in year 2000 (an apparent typing error occurred in Table 1 of Lin and Huybers (2012): the change point *t*_*p*_ should be read as 2000 not 2010). To get a broader picture of this issue, we compared both trends for those crops and traits in the case where data were available on a national level.

Tables 2 and 4 and plots in Figs. 1 and 3 indicate that yield levels for farm harvests are markedly below trial-station yields. Recent relative yield gaps are in the range of 23 % (winter wheat) and 43 % (winter rye). While for winter wheat, spring wheat, spring barley, grain maize and sugar yield the relative magnitude of gap decreased as compared with 1983, the absolute size of gap increased for all crops with rates from 0.17 to 0.76 % p. a. (Tables 2, 4). Fischer et al. (2014) argue that a yield gap of 30 % on farm level, equivalent to about 25 % on trial level (as applied in our study), might be economically attainable, whereas Lobell et al. (2009) set the benchmark to 20 % in developed agriculture. Using the figure of 25 % as the measure, there would be much potential for improvement especially in winter rye, winter triticale and forage maize. Only winter wheat, spring wheat and grain maize are below or at a level of 25 % (Tables 2, 4). The yield gap in winter wheat 2012 of 23 % (Table 2) is equivalent to that of the UK (30 % based on 2008 on-farm) reported by Fischer and Edmeades (2010), indicating about comparable breeding and agronomic conditions. These results raise the question about causes and possible methods to reduce gaps. An intensive discussion of this topic is given, e.g. by Lobell et al. (2009) and Fischer et al. (2014) in chapter 8.

When considering the trend in gap between trial and on-farm yields across all 12 crops, we found significant trend rates in the range of 0.17 % p.a. for spring wheat and 0.76 % p.a. for winter rye (Tables 2, 4, 5). Winter rye provides a noticeable example for diverging trends: the on-farm rate is 0.392 dt ha^−1^ year^−1^ and for trials 0.867 dt ha^−1^ year^−1^, which is more than twice as high. This result indicates that there is in general a strong increase of yield generated by improved new varieties; however, this innovative potential is only partially transferred into higher farm yields.

A closer look at the remarkable differences observed for grain and forage maize reveal that causes may be complex and difficult to substantiate or even to quantify. Results from Table 4 point to high positive genetic, but remarkably negative agronomic trends and contrarily evolving gaps in grain and forage on-farm yield depicting different translation into growing fields. The trial results were obtained from the medium earliness group, whereas on farms not only medium varieties are grown according to the local climatic condition. However, most varieties grown on-farm belong to the medium group, whereas for grain, earlier varieties are used in order to save drying costs, and for forage a shift to later varieties with higher biomass yield took place. In Germany an obvious shift to later maturing varieties was possible due to an increase of higher cold tolerance in the early growing stage, which allowed earlier sowing dates (Volker Klemm, Bundessortenamt, Hannover, personal communication, 2014). As reported by Fischer and Edmeades (2010), US Maize crops in Iowa today are planted 12 days earlier than in 1979. However, those changes are more in favour of higher farm yields and cannot explain the difference between grain and forage maize and zero on-farm yield increase observed in our study. The rather low on-farm rate observed, albeit not significant, may be the effect from several confounded factors. In Germany there was a very rapid extension of maize growing regions towards the north, mainly for biogas production, which doubled its area within the last 10 years. Presently, about 20 % of arable land is grown with maize (see Fig. 5). However, the area extension may not be the only reason, because maize is now not only grown on less fertile soils, but it is now also grown on more fertile fields by replacing, e.g. wheat and sugar beets. It seems necessary to take other factors into account, too. An increasing intensity of maize production leads to closer crop rotation. More soil stress due to heavier farm machinery used to manure fields and to pick up harvest material adds to the problems. Further, rising input prices caused a shift of scarce input resources from forage maize to cash crops, like wheat and winter oilseed rape. In recent years, more emphasis has been laid on breeding of forage maize, which is highly digestible and has high starch content, rather than high fresh matter yield (Wolfgang Schipprack, Universität Hohenheim, personal communication, 9.11.2013). All those complex conditions coming from intensified maize production may be responsible for the low on-farm gain in forage yield. The question arises, if those conditions tend to prevail in variety trials. This is not the case for most factors. In summary the large discrepancy between trial and on-farm yield of forage maize likely arises from changes in management practice.

We checked our data for a plateau effect as reported by Lin and Huybers (2012). The model is described in the Electronic Appendix. The linear-plus-plateau model provided better fit (*p* < 0.01) than the linear regression model for winter wheat (breakpoint *t*_*p*_ = 1999) and winter rye (*t*_*p*_ = 1998) for on-farm yields (Electronic Appendix Table C1; Figure S1 b). In trials only winter wheat showed a plateau effect (*t*_*p*_ = 1997) (Figure S1a). For winter wheat we had data available for 2013 and 2014 and used them to find out, whether the plateau is evolving. However, it turned out, that the linear-plus-plateau model did not provide a significantly better fit (*p* > 0.066) for on-farm yield of both cereals (Electronic Appendix Table C1; Fig. S1c). This result does not support a plateau effect in recent years as far as our data are concerned. A significantly improved fit of a linear-plus-plateau model compared to a linear model does not tell us, if there would be a more realistic non-linear model with better fit. At any rate, random year-to-year variability in yields makes model identification difficult, and a linear trend model is probably good enough for all practical purposes.

The list of factors that commonly affect crop growth and on-farm yield is long and varied (Lobell et al. 2009). The most likely causes for a widening gap between trial-station and on-farm yields in this study may be a shift of acreage to more productive crops, like maize, winter wheat, winter oilseed rape and the decline in legumes, potatoes, oats, barley and winter rye. Further, an agricultural policy which emphasized breeding towards higher factor efficiency and sustainability by reducing fertilizer and pesticide application may have contributed to a widening gap. Rising cost for fertilizer, pesticides, energy and farm machinery and low market prices for cash crops for many years are further reasons for widening the gap.

In order to get sound evidence about the above-mentioned likely causes of diverging yields observed in this study would require more detailed research. Applied levels of fertilizers and agrochemicals, biotic and abiotic factors as well as agronomic practices in both, official trials and on farms, should be assessed from historical data and compared over time.

## Conclusions

We analysed yield progress of 12 important field crops in Germany over the last 30 years, covering 85 % of arable land. The overall yield trend in variety trials was in the range of 0.15 % p.a. for perennial ryegrass total dry matter to 2.04 % p.a. for corrected sugar yield, based on 1983 yield levels. New varieties are the driving force of yield improvement. No decline of genetic progress was observed over time. We showed that for crops other than sugar beets there would have been only moderate increases in yield, for some crops even a decline, if no new varieties had been released. We found significant genetic trends for all crops (except for Italian ryegrass cut 1) covering a wide range of 0.16 % p.a. for dry matter yield of Italian ryegrass up to 1.86 % p.a. for oil yield, while the agronomic trend was between −0.75 % p.a. for forage maize fresh matter yield and 1.12 % p.a. for corrected sugar yield, and mostly not significant. Comparison of treated and untreated cereal trials revealed significant ageing effects. When interpreting genetic and agronomic trends estimated from historical data, we should be aware that rates may be biased if age effects are present.

This study showed that progress in trial yield was transferred only partially to on-farm yield. For all crops even a widening of gap trends was observed. The highest rate of 0.76 % p.a. was found for winter rye. Relative gaps in 2012 are in the range of 23 % for winter wheat and 43 % for winter rye indicating a considerable potential for on-farm yield improvement. Various reasons may be responsible for the apparent gaps, depending on the crop. Shift in acreage, agricultural policy measures and economic reasons to reduce input seem to be of major influence. For the future new improved varieties must continue to be the driving force to generate yield progress. Advanced and locally adapted management technology is needed to translate genetic gain to higher farm yields in order to close yield gaps and to keep in pace with an increasing demand for food needed for a growing world population, and in Germany especially for covering a growing demand for biogas production and renewable resources.

### **Author contributions**

Friedrich Laidig conceived the study and performed all analyses, prepared the figures and wrote large parts of the paper. Hans-Peter Piepho prepared and wrote most of the statistical analysis section and participated in editing the paper. Uwe Meyer compiled all tables. Thomas Drobek and Uwe Meyer assembled all datasets, prepared and formatted them for statistical analysis. Both read and amended the complete manuscript.

## Electronic supplementary material

Below is the link to the electronic supplementary material.

Supplementary material 1 (DOCX 62 kb)
