# Supplementary material for: Genetic and non-genetic long-term trends of 12 different crops in German official variety performance trials and on-farm yield trends
Source: Theor Appl Genet. 2014 Oct 12;127(12):2599–617. doi: 10.1007/s00122-014-2402-z (PMC4236628; doi:10.1007/s00122-014-2402-z)
Supplement: Supplementary file 1 — Supplementary material 1 (DOCX 62 kb) [file 122_2014_2402_MOESM1_ESM.docx]

**Electronic Appendix: Linear-plus-plateau model**

In this Appendix, we apply, in addition to the linear model, a linear-plus-plateau model (Lin and Huybers, 2012) to overall trial and on-farm yields.

**The method:**

- Tested linear-plus-plateau model against linear regression
- This was done by an F-test, which assesses the significance in the increment in the residual sum of squares from a linear to a linear-plus-plateau model
- Linear predictor of linear-plus-plateau model:

  Parameters: $\alpha,\beta,t_{b}$ (breakpoint)
- Fit with some fixed value for $t_{b}$ to get starting values for $\alpha,\beta$
- Nonlinear least squares as implemented in PROC NLIN in SAS 9.4

**The results**:

For on-farm yields, breakpoints were observed in the range of 1991 to 2009 as shown in Table C1. However, only breakpoints for winter wheat (1999) and winter rye (1998) were significant at p < 0.01. Breakpoints of trial yields are closer together (1997-2001), whereas only winter wheat (1997) was significant with p <0.002.

Significance of the test indicates that the linear-plus-plateau model gives a better fit; however, it does not reveal evidence that another model option would be better suited, because year-to-year variability was rather high from 2000 on due to some years with exceptional seasonal growing conditions in Germany. Individual years have considerable influence on the outcome of the test. To demonstrate this, we added results of 2013 and 2014 to winter wheat and winter rye on-farm yield data. In this case the breakpoint shifts to 2001, however, the model test was no longer significant with p > 0.05 (Table C1, Fig. S1).

**Table C1:** Test of linear-plus-plateau model against linear regression and yield gaps. Columns from left to right: year of break point *t_b_*, standard error of estimated break point *se(t_b_)*, probability of model test *p*-value , gaps for harvest years 1983 and 2012. For cereal crops results for intensity 2 are given.

§ Model with breakpoint did not yield a solution with breakpoint inside observed range of years

| Crop | Trait | Break point | | | | | |
| --- | --- | --- | --- | --- | --- | --- | --- |
|  |  | Overall trial yield | | | On-farm yield | | |
|  |  | *t_b_* | *se(t_b_)* | *p*-value | *t_b_* | *se(t_b_)* | *p*-value |
| Winter wheat | Grain yield  (dt ha^-1^) | 1997 | 1.837 | .003 | 1999 | 2.146 | 0.002 |
|  | Grain yield  (dt ha^-1^)^#)^ | - | | | 2000 | 2.175 | 0.066 |
| Winter barley  two rows | Grain yield  (dt ha^-1^) | § | | | 1999 | 3.344 | 0.050 |
| Winter barley  six rows | Grain yield  (dt ha^-1^) | 2001 | 2.867 | 0.196 |  |  |  |
| Winter rye | Grain yield  (dt ha^-1^) | 1998 | 1.655 | 0.1619 | 1998 | 3.491 | .008 |
|  | Grain yield  (dt ha^-1^)^##)^ |  |  |  | 1999.0 | 3.720 | 0.066 |
| Winter triticale | Grain yield  (dt ha^-1^) | 1997 | 2.516 | 0.025 | 1997 | 3.508 | 0.033 |
| Spring wheat | Grain yield  (dt ha^-1^) | 1997 | 4.080 | 0.100 | 1998 | 3.585 | 0.04 |
| Spring barley | Grain yield  (dt ha^-1^) | § | | | 1998 | 3.176 | 0.220 |
| Grain maize | Grain yield  (dt ha^-1^) | 1997 | 4.906 | 0.572 | § | | |
| Forage maize | Total dry matter  (dt ha^-1^) | § | | | 1991 | 5.371 | 0.041 |
| Winter oil seed rape | Grain yield  (dt ha^-1^) | § | | | 2009 | 6.140 | 0.364 |
| Sugar beet | Root yield  (dt ha^-1^) | § | | | § | | |
| Sugar beet | Crude sugar yield  (dt ha^-1^) | § | | | § | | |
| Sugar beet | Sugar yield (corr.)  (dt ha^-1^) | § | | | § | | |

^#)^ Observations for 2013 (80.3 dt/ha) and 2014 (84.3 dt/ha, preliminary estimate) were added

^##)^ Observations for 2013 (59.8 dt/ha) and 2014 (62.7 dt/ha, preliminary estimate) were added


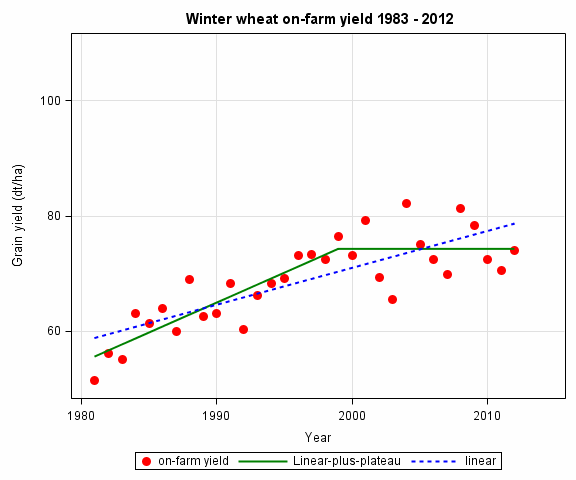

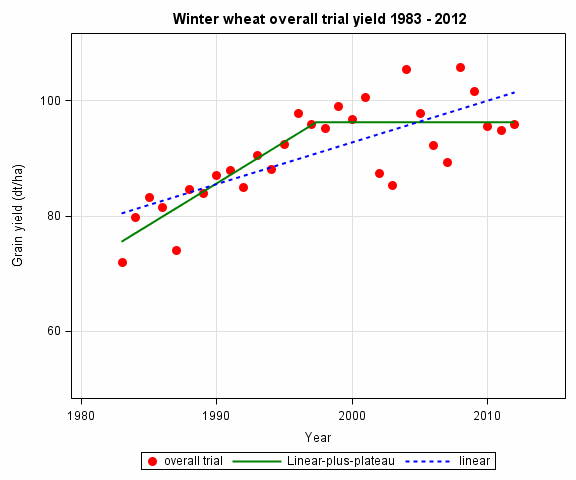
a)


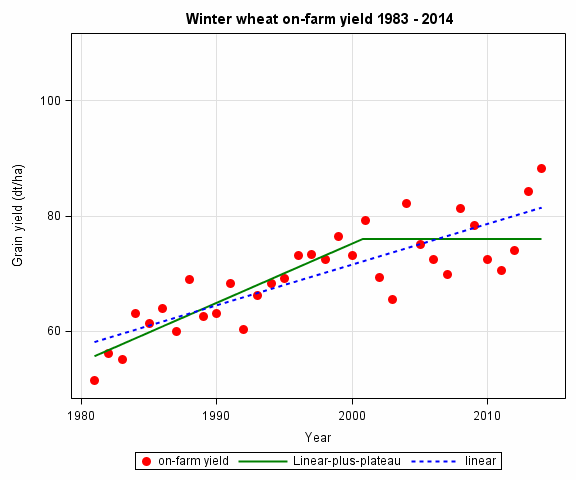
b) c)

**Figure S1:** Linear-plus-plateau model fitted to a) overall trial yields intensity 2, b) on-farm yields, c) on-farm yields by adding observations for 2013 ( 84.3 dt/ha) and 2014 ( 88.3 dt/ha, preliminary estimate).
